# Phenylalanine Stereoisomers of CJ-15,208 and [d-Trp]CJ-15,208 Exhibit Distinctly Different Opioid Activity Profiles

**DOI:** 10.3390/molecules25173999

**Published:** 2020-09-02

**Authors:** Ariana C. Brice-Tutt, Sanjeewa N. Senadheera, Michelle L. Ganno, Shainnel O. Eans, Tanvir Khaliq, Thomas F. Murray, Jay P. McLaughlin, Jane V. Aldrich

**Affiliations:** 1 Department of Pharmacodynamics, The University of Florida, Gainesville, FL 32610, USA; ariana.brice@ufl.edu (A.C.B.-T.); shaieans@cop.ufl.edu (S.O.E.); 2 Department of Medicinal Chemistry, The University of Kansas, Lawrence, KS 66045, USA; nilendrasns@yahoo.com; 3 Torrey Pines Institute for Molecular Studies, Port St. Lucie, FL 34987, USA; condor1082@aol.com; 4 Department of Medicinal Chemistry, The University of Florida, Gainesville, FL 32610, USA; tanvirkhaliq@cop.ufl.edu; 5 Department of Pharmacology and Neuroscience, School of Medicine, Creighton University, Omaha, NE 68178, USA; tfmurray@creighton.edu

**Keywords:** opioid peptide, macrocyclic tetrapeptide, multifunctional ligands, structure-activity relationships, kappa opioid receptor, delta opioid receptor, analgesics, opioid liabilities

## Abstract

The macrocyclic tetrapeptide *cyclo*[Phe-d-Pro-Phe-Trp] (CJ-15,208) and its stereoisomer *cyclo*[Phe-d-Pro-Phe-d-Trp] exhibit different opioid activity profiles in vivo. The present study evaluated the influence of the Phe residues’ stereochemistry on the peptides’ opioid activity. Five stereoisomers were synthesized by a combination of solid-phase peptide synthesis and cyclization in solution. The analogs were evaluated in vitro for opioid receptor affinity in radioligand competition binding assays, and for opioid activity and selectivity in vivo in the mouse 55 °C warm-water tail-withdrawal assay. Potential liabilities of locomotor impairment, respiratory depression, acute tolerance development, and place conditioning were also assessed in vivo. All of the stereoisomers exhibited antinociception following either intracerebroventricular or oral administration differentially mediated by multiple opioid receptors, with kappa opioid receptor (KOR) activity contributing for all of the peptides. However, unlike the parent peptides, KOR antagonism was exhibited by only one stereoisomer, while another isomer produced DOR antagonism. The stereoisomers of CJ-15,208 lacked significant respiratory effects, while the [d-Trp]CJ-15,208 stereoisomers did not elicit antinociceptive tolerance. Two isomers, *cyclo*[d-Phe-d-Pro-d-Phe-Trp] (**3**) and *cyclo*[Phe-d-Pro-d-Phe-d-Trp] (**5**), did not elicit either preference or aversion in a conditioned place preference assay. Collectively, these stereoisomers represent new lead compounds for further investigation in the development of safer opioid analgesics.

## 1. Introduction

The endogenous opioid system is a valuable therapeutic target for the treatment of pain as it is extensively involved in pain perception and experience [1]. The majority of opioid ligands used clinically for the treatment of pain are mu-opioid receptor (MOR) agonists, although agonists of kappa (KOR) and delta (DOR) receptors also produce analgesia. However, opioid-selective agonists also produce a number of undesirable opioid-related side effects that complicate their therapeutic utility. MOR-selective agonists are reinforcing, and produce analgesic tolerance and respiratory depression [2]. In contrast, KOR selective agonists produce dysphoria, sedation, and psychotomimetic effects [3], while DOR-selective agonists can induce seizure activity [4]. 

Multifunctional opioids, ligands with mixed agonist and/or antagonist activity at one or more opioid receptor, have demonstrated potent antinociception, possibly due to synergistic effects [5]. Co-administration of either KOR [6,7] or DOR [8] agonists enhanced the antinociceptive effects of MOR-selective agonists. Some multifunctional opioids also produce reduced side effects [9], a profile attributed to simultaneous modulation of more than one opioid receptor that may counter their individual adverse effects [10]. For example, KOR agonism offsets MOR-mediated reinforcement [11] and respiratory depression [12], while DOR antagonism may slow the development of MOR agonist analgesic tolerance [13,14]. 

**Figure 1:**
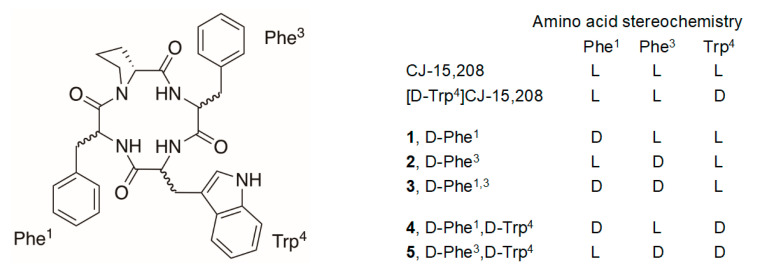
Structures of stereoisomers of CJ-15,208 and [d-Trp]CJ-15,208.

Multifunctional opioid activity has been observed for the structurally distinct macrocyclic tetrapeptide natural product CJ-15,208 (*cyclo*[Phe-d-Pro-Phe-Trp], Figure 1). Originally isolated from the fungus *Ctenomyces serratus*, initial testing found this peptide preferentially bound to KOR and antagonized this receptor in the electrically stimulated rabbit vas deferens [15]. When originally isolated the stereochemistry of the tryptophan residue was not determined, prompting us to synthesize both the l- and d-Trp stereoisomers [16,17]; the optical rotation of the l-Trp isomer was consistent with that reported for the natural product. The two isomeric peptides exhibited similar affinity for opioid receptors (see Table 1) and antagonized KOR in the GTPγS assay in vitro [16,17,18,19]. However, the peptides exhibited distinctly different opioid activity profiles when evaluated in vivo [19]. The d-Trp isomer primarily exhibited KOR antagonism with modest antinociception only at elevated doses, while the l-Trp-containing peptide exhibited mixed, multifunctional activity, with robust antinociception mediated by both KOR and MOR, followed by KOR-selective antagonism lasting several hours after the dissipation of antinociception. Both of these macrocyclic tetrapeptides are active after oral administration [20,21], increasing their potential as leads for drug discovery. 

Therefore, we explored the influence of the stereochemistry of the two phenylalanine residues in CJ-15,208 and [d-Trp]CJ-15,208 (*cyclo*[Phe-d-Pro-Phe-d-Trp]) on opioid activity. All of the stereoisomers retained significant antinociception with reduced liabilities, while the different stereochemistries of the aromatic residues in the five analogs resulted in significant variation in their multifunctional opioid activity.

## 2. Results

### 2.1. Synthesis

The stereoisomers of CJ-15,208 and its d-Trp isomer (Figure 1) were synthesized by a combination of solid phase synthesis of the linear precursors followed by cyclization in solution using modifications to our original strategy [20,22] to improve the yields of the macrocyclic peptides. 

The linear sequences chosen contained the turn inducing D-Pro residue in the middle of the peptide to facilitate cyclization [17], and the use of the 2-chlorotrityl chloride resin minimized the potential for diketopiperazine formation. The peptides were purified by silica gel flash chromatography, which permitted the facile purification of larger quantities of the macrocyclic peptides for in vivo pharmacological evaluation following oral administration. The purified peptides were analyzed by electrospray ionization mass spectrometry, thin layer chromatography, and in two analytical HPLC systems. All of the stereoisomers were obtained in high purity and reasonable yields (34–50% from the linear precursors) after purification.

### 2.2. Metabolic Stability

**Figure 2:**
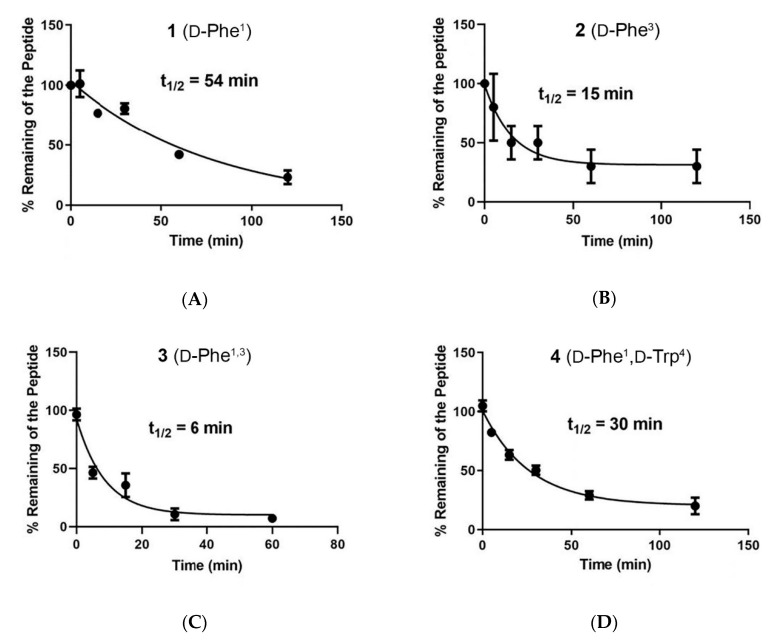

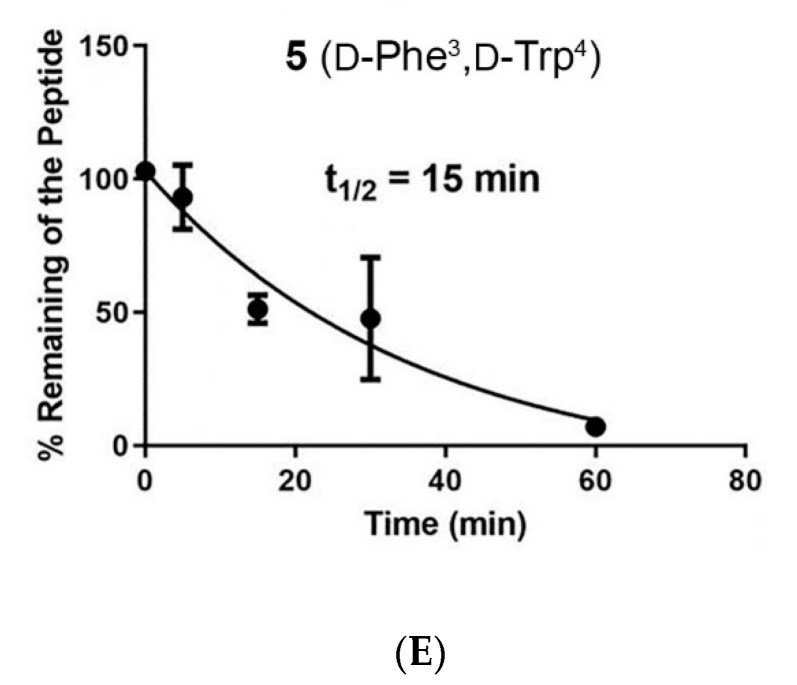
Metabolic stability in mouse liver microsomes of the stereoisomers: (**A**) **1**, (**B**) **2**, (**C**) **3**, (**D**) **4** and (**E**) **5**.

We evaluated the metabolic stability of the stereoisomers in mouse liver microsomes. While macrocyclic peptides are stable to proteases, they can be metabolized by cytochrome P450 enzymes [23,24]. In all cases the peptides were stable in incubations lacking NADPH, but disappeared from the incubations containing NADPH, consistent with cytochrome P450 enzyme metabolism. The half-lives of the stereoisomers in the mouse liver microsomes were ≤30 min for all of the stereoisomers except for **1**, which displayed a half-life more than twice that of most of the other stereoisomers (Figure 2). The short half-lives of most of the stereoisomers are consistent with that of [d-Trp]CJ-15,208 (11 min), while the longer half-life of stereoisomer **1** is similar to that of CJ-15,208 (49 min) (Khaliq et al., manuscript in preparation).

### 2.3. In Vitro Pharmacological Evaluation

In radioligand equilibrium competition binding assays the d-Phe stereoisomers of CJ-15,208 and [d-Trp]CJ-15,208 generally exhibited greatly reduced affinity for KOR and MOR compared to the two parent peptides (Table 1). Only the d-Phe^3^ analogs (see Figure 1 for residue numbering) exhibited sub-micromolar affinity for any of the opioid receptors (K_i_ = ~350 nM for KOR). Similar to CJ-15,208 and [d-Trp]CJ-15,208 [19], the stereoisomers all exhibited negligible affinity for DOR. Also consistent with the results for CJ-15,208 and [d-Trp]CJ-15,208, none of the analogs exhibited appreciable efficacy at either KOR or MOR at 10 μM in [^35^S]GTPγS assays.

### 2.4. In Vivo Pharmacological Evaluation

**Figure 3:**
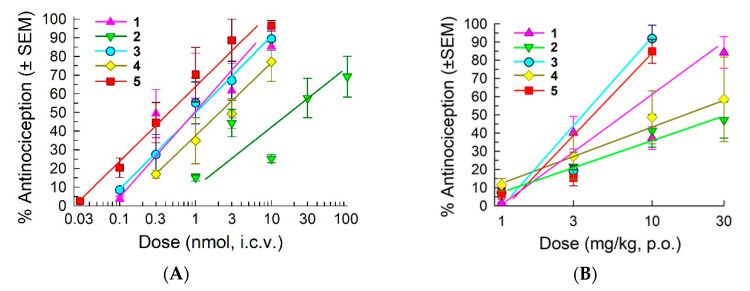
Antinociceptive activity in the 55 °C warm-water tail-withdrawal assay following (**A**) i.c.v. administration and (**B**) oral administration in C57BL/6J mice. All points represent antinociception at peak response, which occurred 20–30 min after administration. Points represent average % antinociception ± SEM from 4–16 mice for each set presented.

The stereoisomers were initially evaluated for their antinociceptive activity in the 55 °C warm-water tail-withdrawal assay in C57BL/6J mice following i.c.v. administration (Figure 3A and Figure S1). All of the d-Phe stereoisomers produced significant time- and dose-dependent antinociception (*p* < 0.05, two-way RM ANOVA). Peak antinociception was produced 20 min after i.c.v administration for stereoisomers **1**, **3**, and **4** and at 30 min for isomers **2** and **5** (Figure S1). The duration of significant antinociception (*p* < 0.05; Dunnett’s *post hoc* test) varied from 45 min (isomers **3** and **4**) to 90 min (isomers **2** and **5**), with isomer **1** exhibiting an intermediate duration. All of the isomers exhibited full and potent antinociception except for **2** (Figure 3A), which produced approximately 70% antinociception at the highest dose tested (100 nmol); the potencies of stereoisomers **1** and **3**–**5** were comparable to that of CJ-15,208 (Table 2). The maximal antinociception found for the stereoisomers **4** and **5** of [d-Trp]CJ-15,208 is in contrast to the parent peptide, which exhibits minimal antinociceptive activity [19].

The d-Phe stereoisomers of CJ-15,208 and of [d-Trp]CJ-15,208 also exhibited significant antinociceptive effects following oral (p.o.) administration (*p* < 0.05, two-way RM ANOVA; Figure 3B and Figure S2). Isomers **1**, **3**, and **5** produced full antinociception with comparable potency to CJ-15,208 (Table 2) that peaked between 20-30 min, while isomers **2** and **4** produced only 45–60% antinociception at the highest oral dose tested (30 mg/kg, p.o.). The duration of significant antinociception (*p* < 0.05, Dunnett’s *post hoc* test) was 50–60 min for all stereoisomers except **4** (30–40 min). While isomer **2** exhibited low antinociceptive efficacy by both routes of administration, isomer **4** exhibited decreased efficacy only following oral administration.

#### 2.4.1. Opioid Receptor Selectivity of Stereoisomer Antinociception

**Figure 4:**
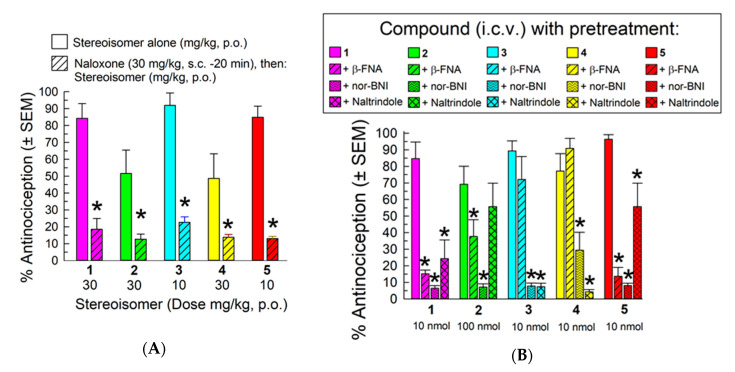
Evaluation of opioid receptor involvement in the antinociceptive activity of the stereoisomers in the 55 °C warm-water tail-withdrawal assay 20 min after (**A**) p.o. administration in mice pretreated with the non-selective opioid receptor antagonist naloxone (30 mg/kg., s.c., −20 min), or (**B**) 20 min after i.c.v. administration in mice pretreated with the selective MOR antagonist β-FNA (10 mg/kg, i.p., −24 h), the selective KOR antagonist nor-BNI (10 mg/kg, i.p., −24 h), or the selective DOR antagonist naltrindole (20 mg/kg, i.p., −20 min). Points represent average % antinociception ± SEM from 8–16 mice for each bar. * significantly different from response of stereoisomer alone (*p* < 0.05. ** two significant bars adjacent to each other.; two-way ANOVA with (**A**) Sidak’s or (**B**) Tukey’s multiple comparisons *post hoc* test).

Pretreatment of mice with an opioid antagonist was used to assess receptor contribution to the observed antinociception, Naloxone (30 mg/kg., s.c.) pretreatment (20 min) significantly reduced the antinociceptive effects of all five analogs (*F*_(4,61)_ = 2.68, *p* < 0.05, two-way ANOVA with Sidak’s *post hoc* test; Figure 4A), consistent with opioid receptors mediating the antinociception. The individual receptor contributions to the observed antinociception were then determined by pretreating the mice with the selective MOR, KOR and DOR antagonists β-FNA (10 mg/kg, i.p., −24 h), nor-BNI (10 mg/kg, i.p., −24 h), or naltrindole (20 mg/kg., i.p., −20 min), respectively, prior to administration of the macrocyclic tetrapeptide (isomers **1**, **3**, **4** and **5** at 10 nmol, and isomer **2** at 100 nmol, i.c.v; Figure 4B). Treatment with these antagonists significantly affected antinociception produced by the stereoisomers (*F*_(12,159)_ = 11.2, *p* < 0.05, two-way ANOVA). β-FNA, nor-BNI, and naltrindole all significantly antagonized the antinociception of stereoisomers **1** and **5** (*p* < 0.05, Tukey’s *post hoc* test), suggesting that all three opioid receptors contributed to the antinociception produced by these stereoisomers. In contrast, isomer **2** demonstrated KOR- and MOR-mediated antinociception, whereas the antinociception produced by **3** and **4** was KOR- and DOR-mediated (*p* < 0.05, Tukey’s *post hoc* test). 

#### 2.4.2. Determination of Stereoisomer Opioid-Receptor Mediated Selective Antagonist Activity

**Figure 5:**
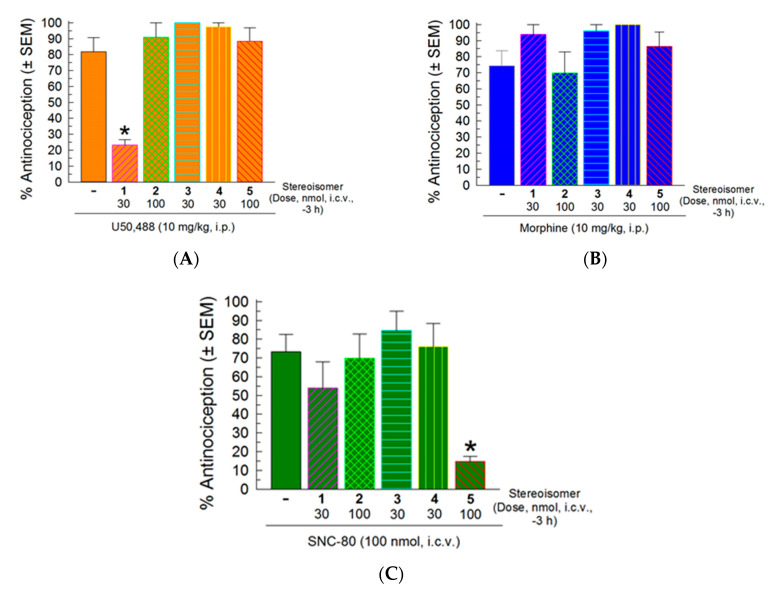
Opioid antagonist activity of the stereoisomers in the 55 °C warm-water tail-withdrawal assay. Mice were pretreated with a stereoisomer (30 or 100 nmol., i.c.v.) 3 h prior to the administration of (**A**) the KOR selective agonist U50,488 (10 mg/kg., i.p.), (**B**) the MOR preferring agonist morphine (10 mg/kg., i.p.), or (**C**) the DOR selective agonist SNC-30 (100 nmol, i.c.v.) to assess their ability to significantly reduce the antinociceptive effect of the opioid agonist. Mean % antinociception ± SEM from 8 mice for each bar. * significantly different from response of agonist alone (*p* < 0.05); one-way ANOVA with Sidak’s multiple comparison *post hoc* test.

Following dissipation of the antinociception, the stereoisomers were evaluated for antagonist activity against the KOR-selective agonist U50,488 (10 mg/kg., i.p.), the MOR-preferring agonist morphine (10 mg/kg, i.p.), and the DOR-selective agonist SNC-80 (100 nmol, i.c.v.; Figure 5). Only isomer **1** (30 nmol, i.c.v.) exhibited significant antagonism of U50,488 (*F*_(5,55)_ = 5.81, *p* < 0.05, one-way ANOVA with Sidak’s *post hoc* test; Figure 5A). Interestingly, stereoisomer **5** (100 nmol, i.c.v) significantly antagonized SNC-80 (*F*_(5,55)_ = 7.71, *p* < 0.05, one-way ANOVA with Sidak’s *post hoc* test; Figure 5C), while none of the peptides demonstrated antagonism against morphine (Figure 5B).

### 2.5. In Vivo Assessment of Opioid Related Liabilities

The five stereoisomers were then assessed for several potential liabilities produced by opioid agonists, specifically impairment of locomotor coordination, respiratory depression, hyperlocomotion and analgesic tolerance.

#### 2.5.1. Assessment of Effects on Coordinated Locomotor Activity

**Figure 6:**
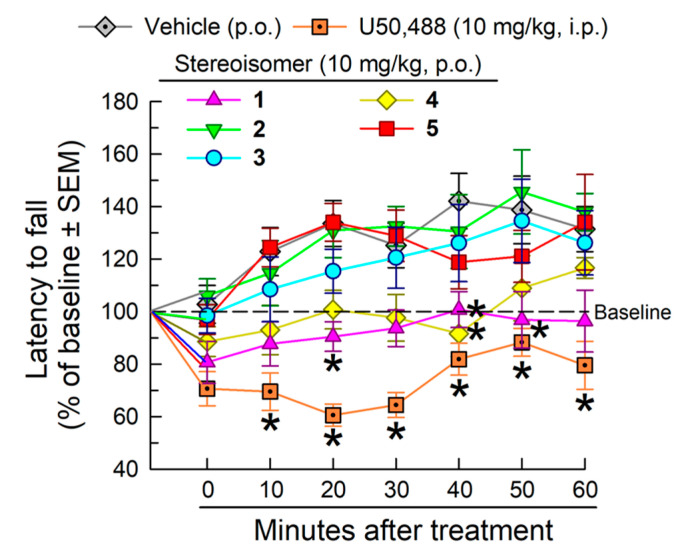
Effect of the stereoisomers on locomotor coordination after p.o. administration to C57BL/6J mice in the rotarod assay. Mice received the macrocyclic tetrapeptide (10 mg/kg, p.o.), vehicle (10% Solutol in saline, p.o.), or U50,488 (10 mg/kg, i.p.) and were tested on the rotarod apparatus with repeated measurements over time. Latencies to fall are given as the mean % change from baseline (100%) performance ± SEM. n = 8 mice per treatment. * significantly different from response of vehicle alone (*p* < 0.05); two-way RM ANOVA with Dunnett’s multiple comparison *post hoc* test.

Treatment with the stereoisomers (10 mg/kg, p.o.) or the KOR-selective agonist U50,488 (10 mg/kg, i.p.) had a significant effect on coordinated locomotor performance in the mouse rotarod assay (*F*_(6,49)_ = 7.91; *p* < 0.05, two-way RM ANOVA) over time (*F*_(6,294)_ = 13.7; *p* < 0.05, two-way RM ANOVA; Figure 6). 

Whereas U50,488 significantly impaired coordinated locomotor activity after the first 10 min compared to vehicle (*p* < 0.05, Dunnett’s *post hoc* test), the stereoisomers **2**, **3** and **5** lacked any significant effect, and isomers **1** (at 20, 40 and 50 min) and **4** (at 40 min) displayed limited impairment of performance (*p* < 0.05, Dunnett’s *post hoc* test).

#### 2.5.2. Evaluation of Respiratory and Spontaneous Locomotor Effects

**Figure 7:**
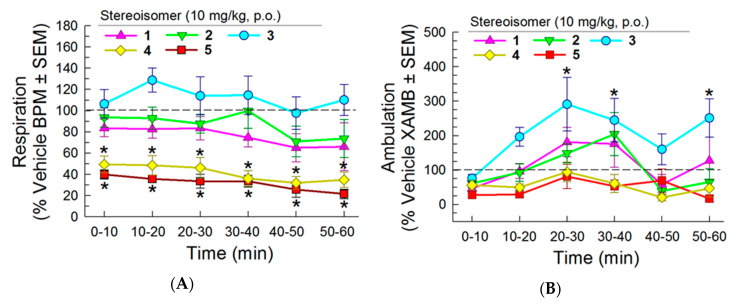
Effects of the stereoisomers on (**A**) respiration and (**B**) ambulation in C57BL/6J mice tested in the CLAMS/Oxymax system. Respiration and ambulation were monitored after administration of stereoisomer (10 mg/kg, p.o.) or vehicle using the CLAMS/Oxymax system. Data from 8–16 mice presented as % vehicle response ± SEM; breaths per minute, BPM (**A**) or ambulation, XAMB (**B**). * significantly different from vehicle control response (*p* < 0.05); two-way RM ANOVA with Dunnett’s multiple comparison *post hoc* test.

The five stereoisomers (10 mg/kg, p.o.) were then assessed for their effect on spontaneous respiration rates and locomotor activity over a 1 h period using the Comprehensive Laboratory Animal Monitoring System (CLAMS) (Figure 7). As expected, the positive control morphine produced significant, time-dependent respiratory depression compared to vehicle (10–30 min; *F*_(10,165)_ = 2.48, *p* < 0.05, two-way RM ANOVA with Dunnett’s multiple comparison *post hoc* test; Figure S2), while treatment with U50,488 resulted in significant, time-dependent increases in respiration rates (20–40 and 50–60 min; *p* < 0.05, Dunnett’s *post hoc* test; Figure S2). Stereoisomers **1–3** of CJ-15,208 did not have any significant effect on respiration compared to vehicle (Figure 7A). Conversely, [d-Trp]CJ-15,208’s isomers **4** and **5** produced significant decreases in respiration rates for the duration of the 60 min testing period (F_(5,50)_ = 12.9, *p* < 0.05, two-way RM ANOVA with Dunnett’s multiple comparison *post hoc* test; Figure 7A). Among the d-Phe stereoisomers, only **3** had a significant effect on ambulation compared to vehicle, demonstrating elevated ambulations at several time-points (20–40 and 50–60 min; *F*_(5,47)_ = 6.20, *p* < 0.05, two-way RM ANOVA with Dunnett’s multiple comparison *post hoc* test; Figure 7B). The positive control morphine produced robust, significant increases in ambulation over the last 40 min of testing (*F*_(10,215)_ = 27.2, *p* < 0.05, two-way RM ANOVA with Dunnett’s multiple comparison *post hoc* test; Figure S2).

#### 2.5.3. Assessment of Acute Antinociceptive Tolerance Development

The stereoisomers were also tested in a model of acute antinociceptive tolerance [25] with repeated dosing (at 0 and 8 h, 0.1–300 nmol, i.c.v.) of morphine, CJ-15,208 or one of the five analogs. The development of acute antinociceptive tolerance was assessed by pretreating with the ED_50_ i.c.v. dose of the test compound, followed 8 h later by treatment with one of a range of graded doses; antinociceptive tolerance was indicated by a significant increase in the ED_50_ value compared to the value observed in naïve animals. As expected, morphine demonstrated acute antinociceptive tolerance, with a significant 7.63-fold rightward shift in the dose-response curve of the second dose administered (*F*_(1,4)_ = 26.2, *p* < 0.05; non-linear regression analysis; Table 3; see also Figure S3). Neither stereoisomer **4** or **5** demonstrated any significant changes in the ED_50_ values collected at 8 h vs. 0 h (Table 3). Both stereoisomers **1** and **3** demonstrated greater acute antinociceptive tolerance than CJ-15,208, with significant rightward shifts in their second dose-response curves (31.2-fold (*F*_(1,5)_ = 39.9 *p* < 0.05) and 5.19-fold (*F*_(1,5)_ = 27.3, *p* < 0.05), respectively; non-linear regression analysis). While isomer **2** demonstrated a 6.13-fold increase in its ED_50_ value after pretreatment, the increase was not statistically significant (*F_(_*_1,4)_ = 2.99, *p* = 0.16.)

#### 2.5.4. Evaluation of Potential Reinforcing or Aversive Properties

**Figure 8:**
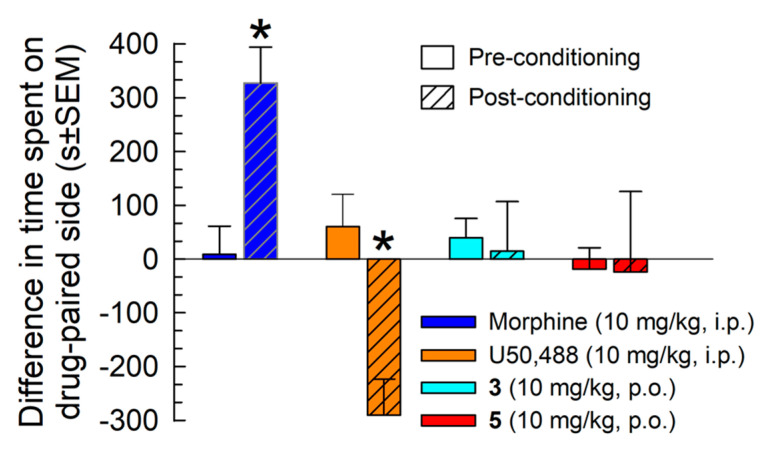
Evaluation of potential rewarding or aversive properties of isomer **3** and **5**. After determination of initial preconditioning preferences, C57BL/6J mice were place conditioned daily for two days with morphine (10 mg/kg, i.p.), U50,488 (10 mg/kg, i.p.), or stereoisomer (10 mg/kg, p.o.) using a counterbalanced design. Data is presented as mean difference in time spent on the drug-paired side ± SEM, with positive and negative values indicating a preference for and avoidance of the drug-paired chamber, respectively. * significantly different from matching preconditioning preference (*p* < 0.05), two-way ANOVA. n = 14–28 mice/compound.

Analogs **3** and **5** were further evaluated in a conditioned place-preference assay (Figure 8). Following a two-day place conditioning paradigm, mice conditioned with morphine (10 mg/kg, i.p.) demonstrated a significant place-preference for the morphine-paired chamber, whereas mice conditioned with U50,488 (10 mg/kg, i.p.) demonstrated a significant conditioned place avoidance (*F*_(3,53)_ = 5.38, *p* < 0.05; two-way ANOVA with Sidak’s multiple comparison *post hoc* test; Figure 8).

However, mice place conditioned with either analog **3** or **5** (10 mg/kg, p.o.) demonstrated no significant preference or aversion for their respective drug-paired chamber.

## 3. Discussion

The macrocyclic tetrapeptide CJ-15,208 is structurally distinct from the endogenous opioid peptides, representing a novel lead compound for the development of new ligands for KOR. Its structure is particularly appealing for modification because its small macrocyclic structure imparts stability to degradation by proteases and facilitates penetration of biological barriers, including both the intestinal and blood-brain barriers [20,21] (Khaliq et al., manuscript in preparation), facilitating systemic, even oral, administration.

Changes in stereochemistry had differential effects on the contributions of the three opioid receptors to antinociception in vivo. While all three aromatic residues of CJ,15-208 were previously found to be important for KOR agonist activity [26], all of the stereoisomers, including the isomers of [d-Trp]CJ-15,208, exhibited KOR agonist activity in vivo regardless of residue stereochemistry. In contrast, only stereoisomer **1** retained KOR antagonist activity. Unlike the parent compounds, all of the stereoisomers except **2** demonstrated antinociception also mediated in part by DOR. This is in contrast to the in vitro results, where the stereoisomers all lacked affinity for DOR in radioligand binding assays; such discrepancies between in vitro and in vivo activity have been found for other CJ-15,208 analogs [26,27,28]. d-Phenylalanine in position 1 appears to favor DOR agonist activity; of the four isomers where DOR contributes to the observed antinociception only **5** does not contain d-Phe^1^, and DOR appears to contribute less to the antinociception of this isomer than for the other isomers (Figure 4B). MOR contributes to the antinociception of three of the stereoisomers, but not to antinociception produced by isomers **3** and **4**. The lack of MOR contribution in these peptides cannot be attributed to the change in stereochemistry of any specific residue; it may instead be related to differences in peptide backbone conformation and the resulting effect on the orientation of one or more of the aromatic residues. Thus only isomer **2** retained the mixed MOR/KOR agonism of CJ-15,208, but this isomer did not exhibit maximum antinociception nor KOR antagonist activity.

All of the stereoisomers retained antinociceptive activity following oral administration, despite the rapid metabolism of most of these peptides by liver microsomes. Such hydrophobic peptides, including the parent peptides (Khaliq et al., manuscript in preparation), generally have high plasma protein binding which can protect compounds from metabolism and clearance, and thereby extend the duration of their activity in vivo. Only in the case of isomer **4** was there a difference in the maximum antinociception following oral vs. central administration. The decreased maximum response and shorter duration following oral administration for this isomer are likely due to its pharmacokinetic properties (metabolism, clearance and/or intestinal absorption).

The stereoisomers exhibited different potential liabilities of use. While KOR agonism contributed to the antinociception of all of the stereoisomers, in the rotarod assay only isomer **5** exhibited significantly decreased locomotor coordination at multiple time points, suggesting that activity at multiple opioid receptors mitigated this known side effect of KOR agonists. The lack of significant decreases in respiratory rates for all three CJ-15,208 isomers was very promising. We have previously shown that the multifunctional macrocyclic tetrapeptide *cyclo*[Pro-Sar-Phe-d-Phe] exhibits reduced liabilities, particularly respiratory effects, and that the peptide’s KOR agonist activity appears to offset respiratory depression mediated by MOR [9]. In contrast, treatment with [d-Trp]CJ-15,208 isomers **4** and **5** decreased respiratory rates; this effect of **4** was surprising, given the lack of MOR agonist activity by this isomer. While not significant, there was a trend towards decreased ambulation following treatment with **4** and **5**, so it is possible that the decreased respiration rates could be due in part to decreased movement by these mice. In contrast to their effects on respiration rates, neither of the [d-Trp]CJ-15,208 stereoisomers exhibited evidence of acute tolerance, while, unlike the parent peptide, the CJ-15,208 isomers exhibited rightward shifts of the dose-response curves to varying degrees. Thus there was a dichotomy between the liabilities observed for the CJ-15,208 stereoisomers, which lacked respiratory depression but exhibited variable acute tolerance, and the [d-Trp]CJ-15,208 isomers, that exhibited the opposite pattern of decreased respiratory rates, but without significant acute tolerance.

Two isomers, **3** and **5**, were selected for testing in the conditioned place-preference assay for reinforcing (conditioned place preference, CPP) or aversive (conditioned place aversion, CPA) effects. Among the CJ-15,208 isomers, **3** demonstrated the most promising activity, producing full antinociception without locomotor impairment or respiratory effects, while the [d-Trp]CJ-15,208 isomer **5** produced full antinociception without acute antinociceptive tolerance. Mice place conditioned with either isomer demonstrated no significant preference or aversion for their respective drug-paired chamber. These results are consistent with earlier tests of multifunctional macrocyclic tetrapeptides [9,19] and could reflect the counteracting effects of agonism at multiple opioid receptors such as MOR and KOR [9,10]. In contrast to **5**, isomer **3** did not demonstrate significant MOR-mediated agonism, but rather KOR- and DOR-mediated antinociception. Mixed action DOR/MOR agonists such as MMP-2200 reportedly do not produce CPP and exhibit limited reinforcing effects [29]. Isomer **5** also displayed DOR antagonism which has been shown to prevent the conditioned place preference of MOR agonists in studies of bivalent ligands [30] and peptidomimetics [31]. To the best of our knowledge, no one has examined the effect of combined KOR agonists/DOR agonists or antagonists. Additional testing of higher doses of **3** and **5** are required to confirm the absence of the place conditioning demonstrated here.

The liabilities of the different stereoisomers did not correlate with the receptor involvement determined in the antinociceptive assay. The antinociception of both the CJ-15,208 stereoisomer **3** and the [d-Trp]CJ-15,208 isomer **4** were mediated by DOR and KOR, without significant contribution from MOR, but these peptides had opposite liability profiles. The same was true for stereoisomers **1** and **5**, where agonist activity mediated by all three opioid receptors contribute to the observed antinociception.

Additional studies will be necessary to explore potential mechanisms for the observed agonist (and in two cases antagonist) activity of these stereoisomers and to better understand receptor contributions to the observed side effects. Such studies are currently ongoing in our laboratories.

The stereoisomer [d-Phe^1,3^]CJ-15,208 (**3**) is a very promising new lead compound for further exploration. Its potent antinociception after oral administration and lack of respiratory depression or locomotor impairment holds significant promise for the identification of safer analgesics.

## 4. Materials and Methods

### 4.1. Chemicals

The sources of the reagents, amino acids, solid phase resin and solvents for peptide synthesis are the same as reported previously [17,26,27]. Amino acids are the l-isomer unless otherwise specified, and abbreviations for amino acids follow the IUPAC-IUB joint commission of biochemical nomenclature (*Eur. J. Biochem.* **1984**, *138*, 9–37). All other chemicals were obtained from Sigma-Aldrich (St. Louis, MO, USA). Thin layer chromatography was performed on glass backed precoated silica gel plates (Sorbent Technologies, Atlanta, GA, USA, or Whatman, aluminum backed, 250 µm layer, Fisher Scientific, Pittsburg, PA, USA), and flash chromatography was performed on standard grade (32–63 μm) silica gel (Sorbent Technologies). HPLC analysis was performed on a Vydac 218TP C18 reversed phase column (Grace Davison, Columbia, MD, USA, 4.6 × 50 mm, 5 µm).

### 4.2. Instruments

Electrospray ionization mass spectra were acquired on a LCT Premier time of flight mass spectrometer (Waters Corp., Milford MA, USA) at the University of Kansas. HPLC analysis was performed using an Agilent 1200 HPLC system (Agilent, Santa Clara, CA, USA).

### 4.3. Peptide Synthesis and Purification

The linear peptide precursors (based on the parent sequences l-/d-Trp-Phe-d-Pro-Phe-OH) were synthesized on a 2-chlorotrityl chloride resin by Fmoc (fluorenylmethoxycarbonyl) peptide synthesis, and the peptides cleaved from the resin with 1% trifluoroacetic acid (TFA) in dichloromethane as described previously [17,26,27]. The crude linear peptides were cyclized using the following general procedure [20,22,27]: The crude linear peptide (0.5 equiv, 21 mM in *N,N*-dimethylformamide, DMF) was added dropwise at a rate of 1.0 mL/h (using a KD Scientific single infusion syringe pump) to a dilute solution of HATU (2-(1H-7-azabenzotriazol-1-yl)-1,1,3,3-tetramethyluronium hexafluorophosphate, 0.75 equiv, 1.2 mM) and *N,N*-diisopropylethylamine (DIEA, 4 equiv, 5 mM) in DMF. After 15 h additional HATU (0.75 equiv) was then added to the reaction in one portion, and additional linear peptide (0.5 equiv, 21 mM in DMF) was added dropwise at a rate of 1.0 mL/h as described above to give a final concentration of the linear tetrapeptide of 2.5 mM. The reaction was then stirred for 6 h at room temperature, followed by an additional 24 h at 37 °C. The solvent was evaporated under reduced pressure, and the crude macrocyclic tetrapeptides isolated as previously described [17,20,26].

The crude peptides were purified by silica gel chromatography using a step gradient of 60–90% EtOAc in hexane (with EtOAc increased in 10% increments), followed by 0–10% MeOH in EtOAc (with MeOH increased in 1% increments). The purified peptides were dissolved in aqueous acetonitrile (water:MeCN, 4:1) and then lyophilized to give the pure peptides as white solids. The purified peptides were analyzed by electrospray ionization mass spectrometry, thin layer chromatography, and in two analytical HPLC systems (see Table 4). The peptides were all >98% pure in both HPLC systems except for **4** which exhibited slightly lower purity. HPLC chromatograms are included in the Supplementary Materials.

#### 4.3.1. [d-Phe^1^]CJ-15,208 (**1**)

Cyclization of the linear peptide (600 mg) according to the general procedure yielded stereoisomer **1** as a white solid (193 mg, 34% yield).

#### 4.3.2. [d-Phe^3^]CJ-15,208 (**2**)

The linear peptide (400 mg) was cyclized according to the general procedure, except that the second addition of peptide was added at a rate of 0.8 mL/h and the final concentration of the linear peptide in the reaction mixture was 4.5 mM. The peptide was purified starting at 60% EtOAc in hexane as described above, followed by 0–30% MeOH in EtOAc (with MeOH increased in 3% increments) to yield stereoisomer **2** as a white solid (167 mg, 43% yield).

#### 4.3.3. [d-Phe^1,3^]CJ-15,208 (**3**)

Cyclization of the linear peptide (600 mg) was performed according to the general procedure above. The purification was performed starting at 50% EtOAc in hexane (with EtOAc increased in 10% increments), followed by 0–5% MeOH in EtOAc (with MeOH increased in 1% increments) to yield **3** as a white solid (294 mg, 50% yield).

#### 4.3.4. [d-Phe^1^,d-Trp^4^]CJ-15,208 (**4**)

The linear peptide (400 mg) was cyclized according to the general procedure above, except that the first addition of peptide was added at a rate of 1.2 mL/h and the final concentration of the linear peptide in the reaction mixture was 1.5 mM. The purification was performed starting at 30% EtOAc in hexane (with EtOAc increased in 10% increments), followed by 0–3% MeOH in EtOAc (with MeOH increased in 1% increments) to yield **4** as a white solid (174 mg, 45% yield).

#### 4.3.5. [d-Phe^3^,d-Trp^4^]CJ-15,208 (**5**)

The linear peptide (430 mg) was cyclized according to the general procedure, except that the second addition of peptide was added at a rate of 0.8 mL/h and the final concentration of the linear peptide in the reaction mixture was 3.6 mM. The peptide was purified starting at 60% EtOAc in hexane as described above, followed by 0–5% MeOH in EtOAc (with MeOH increased in 5% increments) to yield stereoisomer **5** as a white solid (200 mg, 48% yield).

### 4.4. Metabolism by Mouse Liver Microsomes

The macrocyclic tetrapeptide (5 µM) in 1% acetonitrile was incubated with mouse liver microsomes (0.25 mg/mL protein, Xenotech, Lenexa, KS, USA) at 37 ^o^C in the presence or absence of co-factor NADPH RapidStart System (1 mM, Xenotech) or NADPH solution (Sigma, St. Louis, MO, USA) in potassium phosphate buffer (50 mM, pH 7.4). Aliquots (100 µL) taken at 0, 5, 15, 30, 60 and 120 min were quenched with ice-cold acetonitrile (1 vol) containing internal standard ([d-NMeAla^2^]CJ-15,208 [26], 5 µM) to precipitate the proteins. The lack of endogenous interference with the analysis of the stereoisomer or internal standard was confirmed by analyzing samples lacking the macrocyclic tetrapeptide.

Following centrifugation at 10,000× *g* rpm for 10 min, the supernatant (50 µL) was diluted with water (75 µL), stored overnight at −20 °C and analyzed by LC-MS/MS using methodology similar to that described for [d-Trp]CJ-15,208 [32]. Liquid chromatography was performed on a Hypersil BDS C_8_ column (50 mm × 2.1 mm, 3 µm) with a flow rate of 0.2 mL/min and an injection volume of 20 µL using an Acquity UPLC system (Waters, Milford, MA, USA). The peptides were separated using a gradient of aqueous acetonitrile containing 0.08% formic acid of 20% B (0–2 min), 20–50% B (2–3 min), 50–80% B (3–6 min), 80% B (6–7 min), 80–20% B (7–8 min) and 20% B (8–10 min).

ESI-MS/MS was performed on a Waters Quatro triple quadrupole instrument operating in the positive ion multiple reaction monitoring mode. Data acquisition was carried out with Mass Lynx 4.1 software with the following settings: capillary voltage, 2500 V; cone voltage, 30 V; source temperature, 100 °C; desolvation temperature, 250 °C; cone gas flow, 279 L/h; desolvation gas flow, 1157 L/h; LM 1 resolution, 14; HM 1 resolution, 14; ion energy 1, 1.0; MS/MS mode entrance, -1; MS/MS collision energy, 35 eV; MS/MS mode exit, 2; LM 2 resolution, 13.0; HM 2 resolution, 13.0; ion energy 2, 1.5; multiplier, 650; collision cell pressure, 1.63 × 10^−3^ mbar; collision gas, argon. The transition *m*/*z* 578.2 ([M + H]^+^) → 217.2 was monitored to determine the peak area counts of the stereoisomers, and *m*/*z* 566.2 ([M + H]^+^) → 232.9 was monitored to determine the peak area counts of the internal standard (collision energy 22 eV) with the following settings: dwell time, 0.3 s; delay, 0.05 s.

### 4.5. In Vitro Pharmacological Evaluation

Opioid receptor affinities were determined by equilibrium radioligand binding assays as previously described [19,26,33] with membranes from Chinese hamster ovary (CHO) cells stably expressing rat KOR, rat MOR or mouse DOR using the radioligands [^3^H]diprenorphine, [^3^H][d-Ala^2^,*N*-MePhe^4^,glyol]enkephalin (DAMGO) and [^3^H]DPDPE, respectively. Following determination of IC_50_ values by nonlinear regression using Prism software (GraphPad Software Co., La Jolla, CA, USA) K_i_ values were calculated using the Chen and Prusoff equation [34]. The results are presented as the mean ± SEM from at least three separate experiments each performed in triplicate.

Agonist stimulation of [^35^S]GTPγS binding to membranes from CHO cells stably expressing KOR or MOR was assayed as described previously [19,26,35]. The macrocyclic tetrapeptides were screened at 10 µM for efficacy compared to the reference full agonists dynorphin A-(1-13) amide for KOR and DAMGO for MOR. The stereoisomers all exhibited negligible stimulation of GTPγS binding at both KOR and MOR.

### 4.6. In Vivo Testing

#### 4.6.1. Animals and Drug Administration

Adult male wild-type C57BL/6J mice weighing 20–25 g were obtained from Jackson Labs (Bar Harbor, ME, USA). Food pellets and distilled water were available ad libitum. All mice were kept on a 12 h light-dark cycle and were housed and cared for in accordance with the National Institute of Health Guide for the Care and Use of Laboratory Animals. All results of animal testing are reported in accordance with ARRIVE guidelines [36].

For intracerebroventricular (i.c.v.) administration the macrocyclic tetrapeptides were dissolved in dimethyl sulfoxide (DMSO), followed by addition of sterile saline (0.9%) so that the final vehicle was 50% DMSO and 50% saline, and the i.c.v. injections performed as described previously [26]. This concentration of DMSO was not observed to have antinociceptive or behavioral effects in previous use [9,19,21]. For *per os* (p.o.) administration the macrocyclic tetrapeptides were administered in 10% Solutol in 0.9% saline. All solutions for animal administration were prepared fresh daily.

#### 4.6.2. Antinociceptive Testing

The 55 °C warm-water tail-withdrawal assay was performed in mice as previously described [37], with the latency of the mouse to withdraw its tail from the water taken as the endpoint (a cut-off time of 15 sec was used in this assay). Antinociception was calculated according to the following formula: % antinociception = 100 × (test latency − control latency)/(15 − control latency). Tail-withdrawal data points are the means of 8–16 mice, unless otherwise indicated, with SEM shown by error bars.

The opioid receptor involvement in the agonist activity of the macrocyclic peptides was determined by pretreating mice with a single dose of β-funaltrexamine (β-FNA, 10 mg/kg, i.p.) or nor-BNI (10 mg/kg, i.p.) 24 h in advance of administration of a dose of a macrocyclic tetrapeptide. Additional mice were pretreated with a single dose of naloxone (30 mg/kg, s.c.) or naltrindole (20 mg/kg, i.p.) 20 min in advance of administration of the macrocyclic tetrapeptide.

To determine antagonist activity, mice were pretreated with the macrocyclic tetrapeptide 140 min prior to the administration of the MOR-preferring agonist morphine (10 mg/kg, i.p.), KOR-selective agonist U50,488 (10 mg/kg, i.p.) or DOR-selective agonist SNC-80 (100 nmol, i.c.v.); at this time the antinociceptive activity of the stereoisomers had dissipated. Antinociception produced by these established agonists was then measured 40 min after their administration.

#### 4.6.3. Acute Antinociceptive Tolerance Determination

A standardized state of tolerance was induced by administration of morphine or test compound at times 0 and 8 h [25,38,39] to quantitatively evaluate development of acute opioid tolerance. This assay was used to efficiently measure the potential of compounds to cause tolerance using a minimum amount of compound while yielding reliable results. Mice were administered an ED_50_ dose (i.c.v.) of test compound in the morning (time = 0) and a second dose (varying between 0.1–300 nmol, i.c.v.) 8 h later. The degree of tolerance was calculated from the shift in ED_50_ value from the singly- to repeatedly-treated condition [40]. All compounds were administered i.c.v., with antinociception assessed 30 min after injection of morphine or at the time of peak antinociceptive effect of the macrocyclic tetrapeptides, as determined in their initial antinociceptive characterization.

#### 4.6.4. Coordinated Locomotor Activity

The stereoisomers were tested for their possible impairment of locomotor coordination in the rotarod assay as described previously [9,21]. Locomotor activity was recorded using an automated, computer-controlled rotarod apparatus (San Diego Instruments, San Diego, CA, USA). Mice were first habituated to the rotarod over seven trials, with the last trial serving as the baseline response. Mice so habituated were then administered a 10 mg/kg dose of a stereoisomer (p.o.), U50,488 (i.p.), or vehicle 15 min prior to assessment in accelerated speed trials (180 s max latency at 0–20 rpm) performed every 10 min over a 60 min period. Mice were thus tested a total of 14 trials (seven habituation trials prior to treatment + seven drug trials). Decreased latencies to fall in the rotarod test indicate impaired motor coordination/sedation.

#### 4.6.5. Respiration and Ambulation

Respiration rates (in breaths per minute) and animal locomotive activity (as ambulations) were assessed using the Oxymax/CLAMS system (Columbus Instruments, Columbus, OH, USA) as described previously [9,25]. Mice were habituated to their individual sealed housing chambers for 60 min before testing. Mice were administered stereoisomer (10 mg/kg, p.o.), morphine (10 mg/kg, i.p.), U50,488 (10 mg/kg, i.p.), or vehicle, as indicated, and five min later confined to the CLAMS testing chambers. Pressure monitoring within the sealed chambers measured frequency of respiration. Infrared beams located in the floor measured locomotion as number of beam breaks. Respiration and locomotive data were averaged over 10 min periods for 60 min post-injection of the test compound. Data is presentenced as % vehicle response ± SEM, ambulation or breaths per minute.

#### 4.6.6. Evaluation of Potential Conditioned Place Preference and Conditioned Place Aversion

An automated, balanced three-compartment place conditioning apparatus (San Diego Instruments, San Diego, CA, USA) and a 2-day counterbalanced place conditioning design was used similar to methods previously described [21]. The amount of time subjects spent in each of the three compartments was measured over a 30 min testing period. Prior to place conditioning an initial preference test was performed in which the animals could freely explore all open compartments; the animals did not demonstrate significant differences in their time spent exploring the outer left versus right compartments (*p* > 0.05, Student’s *t*-test). For place conditioning mice were administered 0.9% saline (i.p.) and consistently confined in a randomly assigned outer compartment: half of each group in the right chamber, and half in the left chamber. Four hours later, mice were administered test compound and confined to the opposite compartment for 40 min. To determine if **3** or **5** (10 mg/kg, p.o.) produced CPP or CPA, mice were place conditioned in this way for two days, with a final preference test taken on the fourth day, as this has been shown to produce dependable morphine CPP and U50,488-induced CPA [41]. Additional groups of mice were placed conditions with morphine or U50,488 (10 mg/kg, i.p.) as positive controls.

### 4.7. Statistical Analysis

All dose-response lines were analyzed by regression, and ED_50_ (effective dose producing 50% antinociception) values and 95% confidence intervals (C.I.) determined using individual data points from graded dose-response curves with Prism 8.0 software (GraphPad, La Jolla, CA, USA). Percent antinociception was used to determine within group effects and to allow comparison to baseline latency in tail-withdrawal experiments. The statistical significance of differences between ED_50_ values was determined by evaluation of the ED_50_ value shift via nonlinear regression modeling with Prism software. Significant differences in behavioral data were analyzed by ANOVA (one-way or two-way with repeated measures (RM), as appropriate). Significant results were further analyzed with Sidak’s, Tukey’s, or Dunnett’s multiple comparison *post hoc* tests, as appropriate. Data for conditioned place preference experiments were analyzed by two-way RM ANOVA, with analyses examining the main effect of conditioned place preference phase (e.g., pre- or post-conditioning) and the interaction of drug pretreatment. Significant effects were further analyzed using Sidak’s HSD *post hoc* testing. All data are presented as mean ± SEM, with significance set at *p* < 0.05.

## 5. Patents

J.V. Aldrich and S. Senadheera, Cyclic Tetrapeptide Stereoisomers, U.S. Patent 10,259,843 B2, 2019, and European patent EP3,166,625, 23019.

## Figures and Tables

**Table 1:** Opioid receptor affinities of the stereoisomers of CJ-15,208 and [d-Trp]CJ-15,208 ^a,b^.

Stereoisomer	K_i_ (nM ± SEM)		Selectivity
	KOR	MOR	K_i_ (MOR) K_i_ (KOR)
**1** (d-Phe^1^)	5120 ± 690	>10,000	<2
**2** (d-Phe^3^)	362 ± 51	3920 ± 200	11
**3** (d-Phe^1,3^)	2560 ± 480	7780 ± 410	3
CJ-15,208	27.4 ± 4.6	451 ± 114	16.5
**4** (d-Phe^1^, d-Trp^4^)	>10,000	>10,000	-
**5** (d-Phe^3^, d-Trp^4^)	353 ± 19	5800 ± 1450	16
[d-Trp^4^]CJ-15,208	21.8 ± 4.8	259 ± 29	12

^a^ Data are the mean K_i_ values ± SEM from at least three experiments. ^b^ None of the stereoisomers at 10 µM exhibited appreciable affinity for DOR (<30% inhibition of [^3^H]*cyclo*[d-Pen^2^,d-Pen^5^]enkephalin (DPDPE) binding).

**Table 2:** Summary of in vivo opioid antinociceptive activity of the stereoisomers ^a^.

Stereoisomer	ED_50_ (and 95% Confidence Interval (C.I.)) Values		
	i.c.v. (nmol)	p.o. (mg/kg)	Receptors Involved
**1**	0.75 (0.36–1.44)	7.62 (5.12–12.2)	KOR, MOR, DOR
**2**	20.4 (10–58.7)	~	KOR, MOR
**3**	1.00 (0.64–1.60)	4.12 (3.30–5.31)	KOR, DOR
CJ-15,208 ^b,c^	1.74 (0.62–4.82)	3.49 (1.98–5.73)	KOR, MOR
**4**	2.39 (1.40–4.56)	~	DOR, KOR
**5**	0.56 (0.38–0.91)	4.72 (3.70–6.39)	KOR, MOR, DOR
[d-Trp^4^]CJ-15,208 ^b,d^	~	~	-

^a^ In addition, isomer **1** exhibited antagonist activity at KOR, and isomer **5** exhibited antagonist activity at DOR. ^b^ Ref. [19]; ^c^ Ref. [20]; ^d^ Ref. [21]. ~ Maximum antinociception not achieved, precluding calculation of an ED_50_ value.

**Table 3:** Comparison of ED_50_ (and 95% C.I.) values in naïve subjects and after again 8 h after a treatment of an ED_50_ dose of the respective compound ^a^.

Stereoisomer	Naïve ED_50_ (95% C.I.)	ED_50_ (95% C.I.) Pretreated Mice	Fold-Shift, Naïve ED_50_ vs. Second ED_50_
**1**	0.65 (0.31–1.31)	20.3 * (11.1–41.7)	31.2
**2**	20.4 (10–58.7)	125 (89.9–224)	6.13
**3**	1.00 (0.64–1.60)	5.19 * (2.57–11)	5.19
**4**	2.39 (1.40–4.56)	1.28 (0.96–1.66)	0.54
**5**	0.45 (0.28–0.70)	0.49 (0.09–1.36)	1.09
CJ-15,208	1.82 (1.22–2.74)	5.23 (4.23–6.48)	2.87
Morphine	2.91 (2.48–3.41)	22.2 * (14.0–55.3)	7.63

^a^ Data are ED_50_ values (nmol) from C57BL/6J mice tested with one of several doses (i.c.v.) of compound in the 55 °C warm-water tail-withdrawal assay in either naïve animals or mice who were pretreated with the ED_50_ dose of the respective compound, followed by administration of varying doses 8 h later. * significantly different from ED_50_ value in naïve mice (*p* < 0.05), non-linear regression analysis. n = 8–16 mice/dose tested.

**Table 4:** Analytical data for stereoisomers of CJ-15,208 and [d-Trp]CJ-15,208.

	Observed	TLC		HPLC, t (min)
Isomer	ES-MS, *m*/*z* ^a^	R_f_^b^	System A ^c^	System B ^d^
**1**	600.2568	0.70	18.4	23.0
**2**	600.2565	0.10	18.9	–
**3**	600.2578	0.67	22.4	22.6
**4**	600.2584	0.49	18.6 ^e^	21.9 ^e^
**5**	600.2605	0.53	21.1	23.8

^a^ M + Na^+^, calculated *m*/*z* 600.2587; ^b^ EtOAc: MeOH, 9:1; ^c^ 15–55% MeCN over 40 min with 0.1% TFA, detection at 214 nm; ^d^ 30–70% MeOH over 45 min with 0.1% TFA, detection at 230 nm. ^e^ 94% and 96% pure in systems A and B, respectively.

## Supplementary Materials

The following are available online, Figure S1: Time-course of antinociceptive activity in the 55 °C warm-water tail-withdrawal assay following (A) i.c.v. administration and (B) oral administration in C57Bl/6J mice of a maximally efficacious dose. Points represent average % antinociception ± SEM from 4–16 mice for each set presented, Figure S2. Effects of the U50,488 or morphine on (A) respiration and (B) ambulation in C57BL76J mice. Respiration and ambulation were monitored after administration of U50,488 or morphine (10 mg/kg, i.p.) using the CLAMS/Oxymax system. Data from 9–18 mice presented as % vehicle response ± SEM; breaths per minute, BPM (A) or ambulation, XAMB (B). * significantly different from response of saline alone (*p* < 0.05); two-way RM ANOVA with Dunnett’s multiple comparison post hoc test., Figure S3. Evaluation of acute antinociceptive tolerance in the 55 °C warm-water tail-withdrawal assay following i.c.v. administration of morphine, (A) CJ-15,208, (B) **1**, (C) **2**, (D) **3**, (E) **4** or (F) **5**. All points represent antinociception at peak response in naïve mice (Time 0 h) and mice that were previously administered an ED_50_ dose of test compound (as listed) prior to additional administration of a graded dose of test compound eight hours later (Time 8 h). Points represent average % antinociception ± SEM from 8–16 mice for each set presented, Figure S4. HPLC chromatograms of the peptides in 15–55% MeCN over 40 min with 0.1% TFA, detection at 214 nm, (A) **1**, (B) **2**, (C) **3**, (D) **4** and (E) **5,** Figure S5. HPLC chromatograms of the peptides in 30–70% MeOH over 40 min with 0.1% TFA, detection at 230 nm, (A) **1**, (B) **3**, (C) **4** and (D) **5**.
