# Supplementary material for: Phenylalanine Stereoisomers of CJ-15,208 and [d-Trp]CJ-15,208 Exhibit Distinctly Different Opioid Activity Profiles
Source: Molecules. 2020 Sep 2;25(17):3999. doi: 10.3390/molecules25173999 (PMC7504817; doi:10.3390/molecules25173999)
Supplement: Supplementary file 1 [file molecules-25-03999-s001.pdf]

Supplementary Material

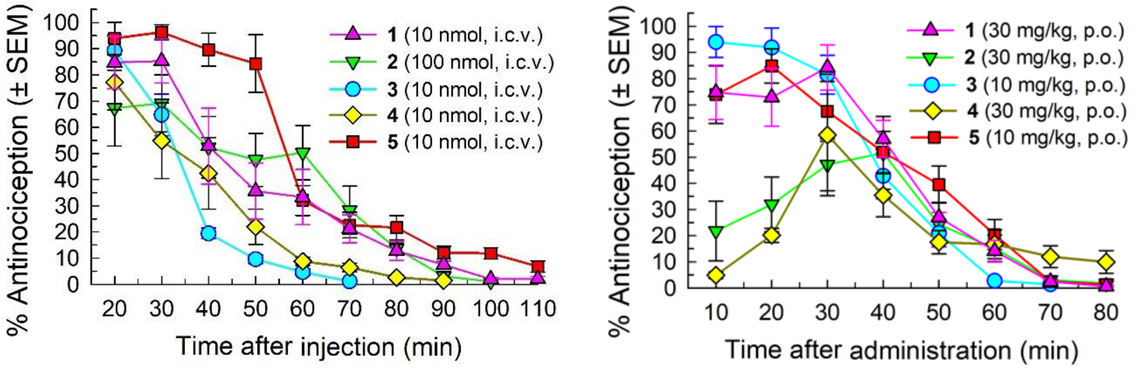

(**A**) (**B**)

Figure S1. Time-course of antinociceptive activity in the 55 °C warm-water tail-withdrawal assay following (**A**) i.c.v. administration and (**B**) oral administration in C57Bl/6J mice of a maximally efficacious dose. Points represent average % antinociception ± SEM from 4-16 mice for each set presented.

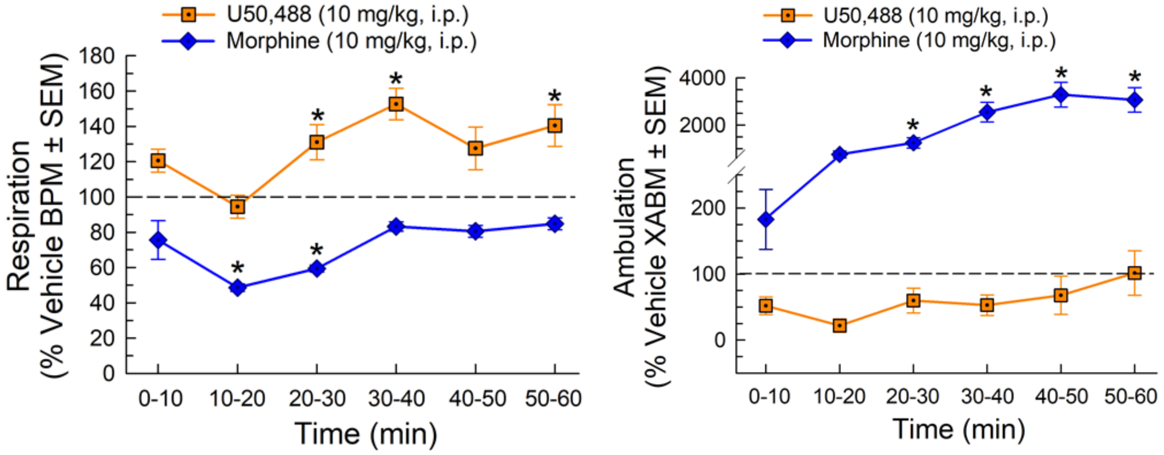

(**A**) (**B**)

Figure S2. Effects of the U50,488 or morphine on (**A**) respiration and (**B**) ambulation in C57BL76J mice. Respiration and ambulation were monitored after administration of U50,488 or morphine (10 mg/kg, i.p.) using the CLAMS/Oxymax system. Data from 9-18 mice presented as % vehicle response ± SEM; breaths per minute, BPM (**A**) or ambulation, XAMB (**B**). *significantly different from response of saline alone ($p < 0.05$); two-way RM ANOVA with Dunnett's multiple comparison *post hoc* test.

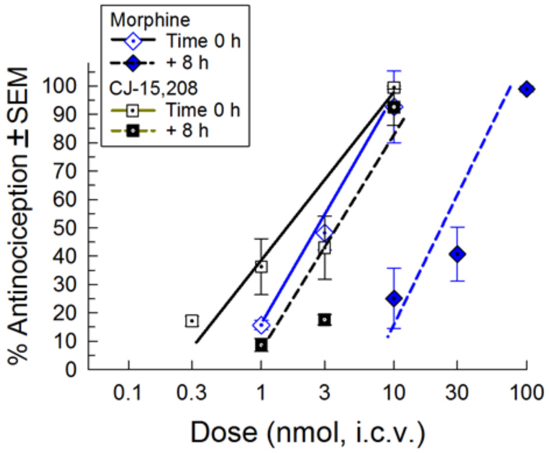
Morphine
Time 0 h
+ 8 h
CJ-15,208
Time 0 h
+ 8 h
% Antinociception ± SEM
0
10
20
30
40
50
60
70
80
90
100
0.1
0.3
1
3
10
30
100
Dose (nmol, i.c.v.)

(**A**)

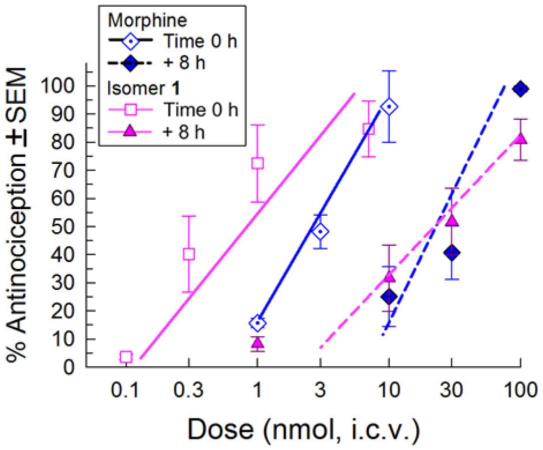
Morphine
Time 0 h
+ 8 h
Isomer 1
Time 0 h
+ 8 h
% Antinociception ± SEM
0
10
20
30
40
50
60
70
80
90
100
0.1
0.3
1
3
10
30
100
Dose (nmol, i.c.v.)

(**B**)

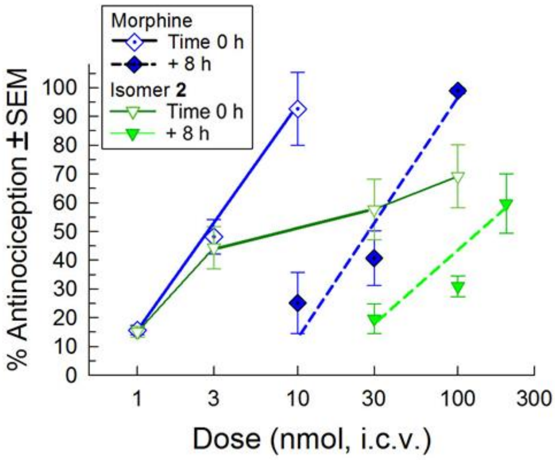
Morphine
Time 0 h
+ 8 h
Isomer 2
Time 0 h
+ 8 h
% Antinociception ± SEM
0
10
20
30
40
50
60
70
80
90
100
1
3
10
30
100
300
Dose (nmol, i.c.v.)

(**C**)

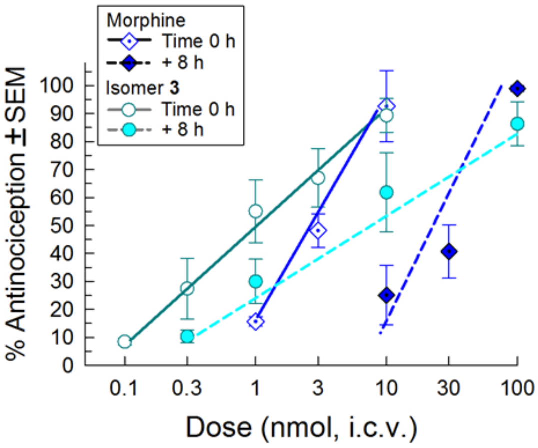
Morphine
Time 0 h
+ 8 h
Isomer 3
Time 0 h
+ 8 h
% Antinociception ± SEM
0
10
20
30
40
50
60
70
80
90
100
0.1
0.3
1
3
10
30
100
Dose (nmol, i.c.v.)

(**D**)

Figure S3. *Cont.*

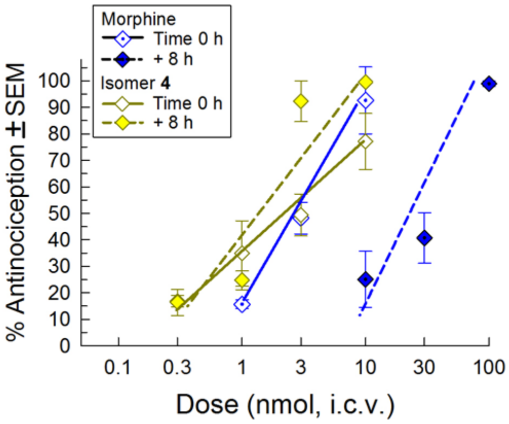

(**E**)

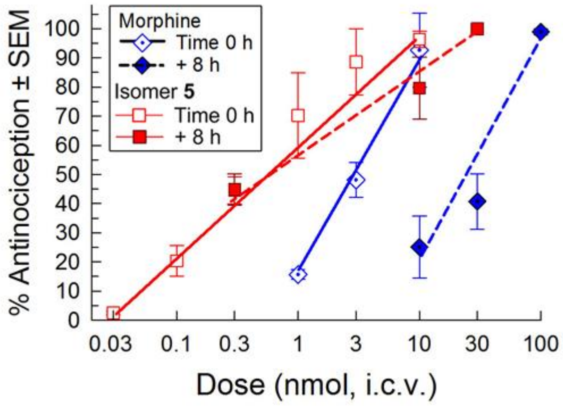

(**F**)

Figure S3. Evaluation of acute antinociceptive tolerance in the 55 °C warm-water tail-withdrawal assay following i.c.v. administration of morphine, (**A**) CJ-15,208, (**B**) **1**, (**C**) **2**, (**D**) **3**, (**E**) **4** or (**F**) **5**. All points represent antinociception at peak response in naïve mice (Time 0 h) and mice that were previously administered an ED_{50} dose of test compound (as listed) prior to additional administration of a graded dose of test compound eight hours later (Time 8 h). Points represent average % antinociception ± SEM from 8-16 mice for each set presented.

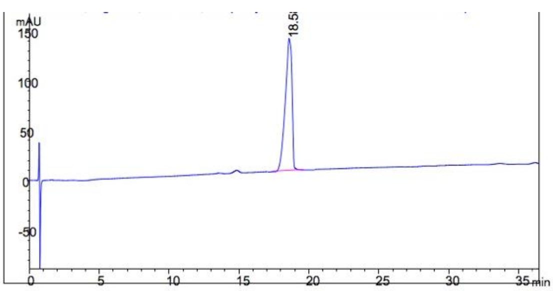

(**A**)

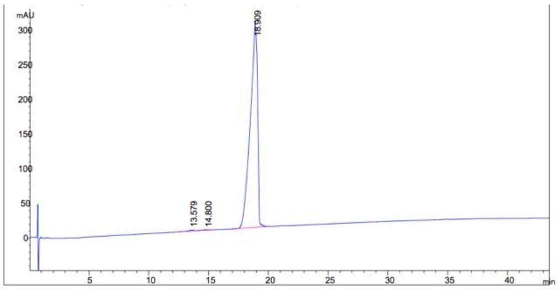

(**B**)

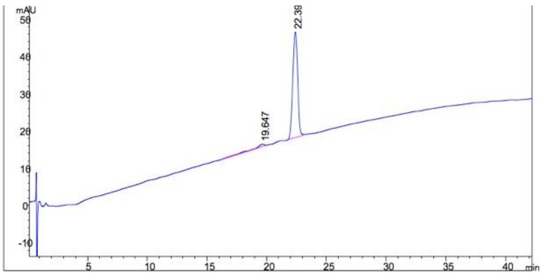

(**C**)

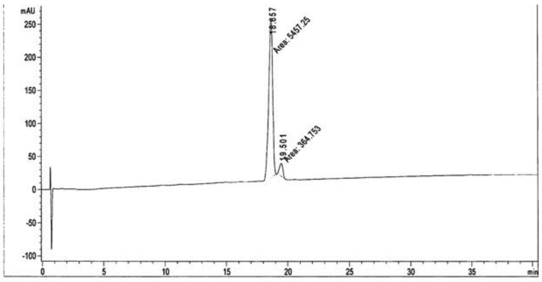

(**D**)

Figure S4. *Cont.*

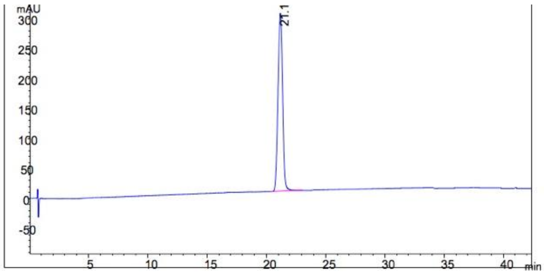

(**E**)

Figure S4. HPLC chromatograms of the peptides in 15–55% MeCN over 40 min with 0.1% TFA, detection at 214 nm, (**A**) **1**, (**B**) **2**, (**C**) **3**, (**D**) **4** and (**E**) **5**

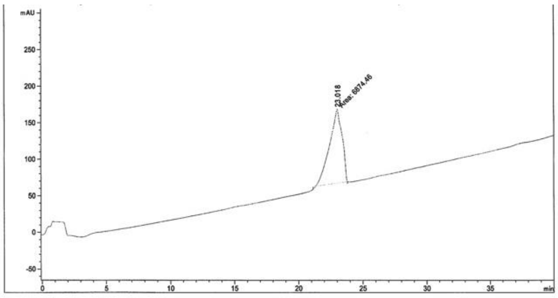

(**A**)

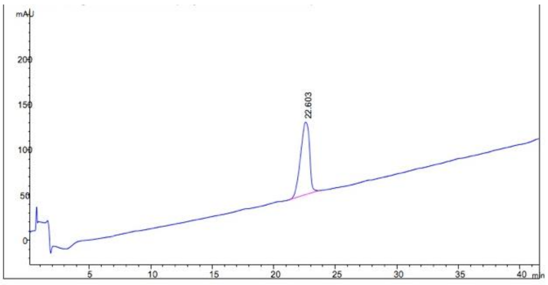

(**B**)

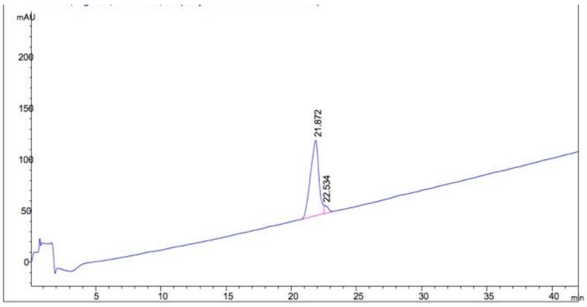

(**C**)

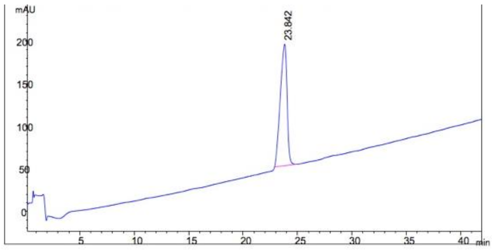

(**D**)

Figure S5. HPLC chromatograms of the peptides in 30–70% MeOH over 40 min with 0.1% TFA, detection at 230 nm, (**A**) **1**, (**B**) **3**, (**C**) **4** and (**D**) **5**
